# arcMS: transformation of multi-dimensional high-resolution mass spectrometry data to columnar format for compact storage and fast access

**DOI:** 10.1093/bioadv/vbae160

**Published:** 2024-10-26

**Authors:** Julien Le Roux, Julien Sade

**Affiliations:** LEESU, Univ Paris Est Créteil, Ecole des des Ponts, Créteil F-94010, France; LEESU, Univ Paris Est Créteil, Ecole des des Ponts, Créteil F-94010, France

## Abstract

**Summary:**

The arcMS R package addresses the challenges posed by proprietary and open-source high-resolution mass spectrometry data formats by providing functions to collect MS^E^ data from the Waters UNIFI software and store it in the efficient Apache Parquet format, facilitating fast, easy access, and compatibility with various programming environments. This solution facilitates the manipulation of complex mass spectrometry data, including ion mobility or other additional dimensions, enabling potential integration into efficient and open-source workflows.

**Availability and implementation:**

arcMS is an open-source R package and is available on GitHub at https://github.com/leesulab/arcMS. The complete documentation, including details on UNIFI configuration and tutorials for data conversion, access to Parquet files, and filtration of data, is available at https://leesulab.github.io/arcMS. An R/Shiny companion application is also provided for visualization of Parquet data and demonstration of data filtering with the Arrow library https://github.com/leesulab/arcms-dataviz.

## 1 Introduction

Many file formats are employed for storing and processing mass spectrometry data using either open-source software or proprietary commercial products (Deutsch 2012). Proprietary formats specific to instrument vendors are often used for long-term data storage due to their high compression rates in binary format and compatibility with the vendors’ software. However, these formats lack interoperability and flexibility, hindering data integration into customized workflows, especially for users working with multiple vendors’ equipment or open-source software. Most vendors provide the ability to convert data to open formats with tools such as ProteoWizard (Chambers et al. 2012) or offer direct access to raw data through APIs. In particular, Waters high-resolution mass spectrometry (HRMS) instruments store data in an Oracle database, which can be accessed via APIs provided with the instrument’s software (UNIFI or waters_connect). While open formats like mzML (Martens et al. 2011) facilitate data exchange and standardization, they often result in large file sizes and poor random access performance, especially with HRMS data. Other formats (e.g. mz5, mzMLb) offer more efficient compression but still lack compatibility with most open-source software, ease of use, and fast random access (Bhamber et al. 2021, Wilhelm et al. 2012). Advancements in HRMS instruments, including the integration of additional separation dimensions such as ion mobility spectrometry (IMS), have led to increased data volumes and complexity. Converting data acquired with IMS can produce very large files, often several gigabytes each. Various open-source software and workflows have been developed to analyze data from open formats [e.g. MS-DIAL (Tsugawa et al. 2015), MZmine (Schmid et al. 2023), XCMS (Smith et al. 2006), openMS (Pfeuffer et al. 2017), Workflow4Metabolomics (Guitton et al. 2017)]. In environmental science, specific open tools are also available to perform non-target screening of pollutants [e.g. patRoon (Helmus et al. 2021), NORMAN Suspect List Exchange (Mohammed Taha et al. 2022)]. However, most of these tools lack compatibility with data acquired using IMS. The MZmine 3 platform recently added support for multi-dimensional data from various instrumental setups, including IMS, but it requires mzML files as input data.

### 1.1 arcMS and the Apache Parquet format

The arcMS package addresses the storage challenges of traditional HRMS file formats and the limitations of processing mzML files containing IMS data, e.g. from UPLC-IMS-QTOF analytical systems. It is specifically developed to convert data acquired in MS^E^ mode (Data-Independent Analysis, with or without IMS) from the Waters UNIFI software, facilitating efficient spectral data retrieval through the Waters UNIFI (or waters_connect) API. Data are converted to the Apache Parquet format, renowned for its compactness and efficiency in handling large datasets. Parquet’s columnar structure is optimized for efficient storage and retrieval of complex, nested data structures, offering high compression ratios and efficient encoding methods. Moreover, the Parquet format is an open-source, language-agnostic format compatible with various programming languages (e.g. R, Python, Julia, Java), enabling users to access their HRMS data within their preferred programming ecosystem.

## 2 Methodology

### 2.1 Package design

Developed in the R language, arcMS provides functions and a Shiny web app to easily connect to the UNIFI database, navigate the folder structure, retrieve data, and perform conversion. Asynchronous HTTP requests to the UNIFI API are executed using the future framework (Bengtsson 2021) for fast spectral data collection. The data are received as a Protocol Buffers binary message, deserialized using the RProtoBuf package (Eddelbuettel et al. 2016), and structured into a simple tabular dataframe. Data are saved in the Apache Parquet format, with associated metadata stored both in JSON format and within the Parquet file metadata. This approach ensures very fast random access to the data and easy filtration of spectra, along with a small file size.

### 2.2 Data structure in the Parquet format

HRMS data are structured as a long-format tabular dataframe (Table 1). The dataframe includes typical spectral dimensions (scan ID, retention time, *m/z* ratio, and intensity columns) as well as additional dimensions related to IMS, if present (bin index and corresponding drift time), for both MS1 and MS2 levels (low and high collision energy in HDMS^E^ mode). This tabular format facilitates quick data filtration (e.g. by MS level or to obtain extracted ion chromatograms within a specific *m/z* range) and ensures easy integration with other existing pipelines using the same data structure, such as the DEIMoS Python library (Colby et al. 2022).

**Table 1.** Data structure example of the dataframe obtained from arcMS and stored in the Parquet format.^a^

rt	scanid	mslevel	mz	intensity	bin	dt
0.0074	1	1	105.9348	57	39	2.769
0.0074	1	1	128.9497	123	35	2.485
0.0116	1	2	172.8606	19	60	4.260
0.0158	2	1	87.9227	14	40	2.840
0.0241	3	1	128.9497	96	34	2.414

a For HDMS^E^ data, low and high collision energy spectra are attributed to MS levels 1 and 2, respectively. rt, retention time; scanid, ID number of a scan (repeated for each bin index/drift time at the same retention time and same MS level); mz, *m/z* ratio; dt, ion mobility drift time.

### 2.3 Testing and validation

Validation tests were conducted using environmental data acquired from a UPLC-IMS-QTOF system (Waters Vion). Data generated by arcMS and saved in Parquet format were compared with open formats (mzML, mz5, and mzMLb) obtained using ProteoWizard MSConvertGUI version 3.0.24170 (Holman et al. 2014). Conversion with MSConvertGUI utilized 64-bit binary encoding precision, zlib compression, no numpress compression, and optional gzip compression. Raw data were converted in profile mode without applying any filter, i.e. without any peak picking. To evaluate conversion speed, three samples were converted in triplicate using both arcMS and ProteoWizard on the same computer than the UNIFI database (Windows 10, Intel Xeon W-2125 4.0 GHz, 64 GB RAM). Storage space requirements for different formats were assessed across 10 samples, including 2 blank, 5 calibration (mix of analytical standards), and 3 environmental samples (surface water impacted by wastewater discharge). Data reading speed was tested on three samples, each repeated three times, measuring access time in a Python environment. Parquet files were opened directly as DataFrames using the pandas read_parquet() function, while mzML files (with and without gzip compression) were opened using the DEIMoS library (with the deimos.load() function), both producing DataFrames usable by DEIMoS.

## 3 Performance evaluation

The fidelity of the conversion process was assessed by comparing spectra of known compounds (analytical standards) in the UNIFI software interface with spectra obtained after conversion to various formats, all showing exact matches. Python DataFrames obtained from mzML files using the DEIMoS library and DataFrames obtained directly from the Parquet files created by arcMS were also found to be identical.

**Figure 1.**
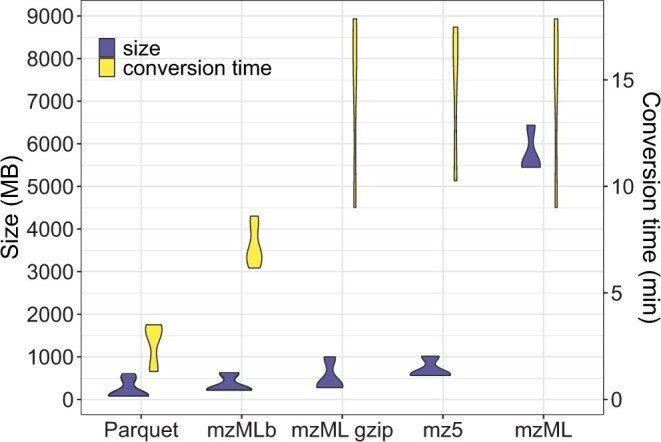
File sizes and conversion duration for 10 different samples (blanks and environmental samples) in various data formats. Parquet files were obtained with arcMS, other formats were obtained with ProteoWizard MSConvertGUI.

Performance comparisons in terms of conversion speed, file size, and data read speed highlighted the efficiency of the Parquet format relative to other formats. Processing a set of 10 HRMS samples with arcMS took between 1 and 3 min, whereas MSConvertGUI required between 11 and 18 min to collect and convert data to mz5, mzML, or gzipped mzML formats (Fig. 1). Conversion to mzMLb files took ∼7 min.

Regarding file sizes, Parquet files were the smallest, ranging from 80–100 MB for clean samples (blanks, calibration samples) to 400–600 MB for complex environmental samples. In contrast, uncompressed mzML files reached 5–6 GB, while gzipped mzML files and mz5 format varied between 300–600 MB for clean samples and 1 GB for complex samples (Fig. 1). Only mzMLb files reached similar sizes to Parquet files for environmental samples (∼600 MB), but clean samples showed larger file sizes (∼200 MB). Gzip compression is also possible with the mz5 format, further reducing file sizes to those obtained with the mzMLb format (200–600 MB). The size of Parquet files appeared very dependent on the number of spectra present in the raw data, achieving minimal sizes for samples with fewer peaks, while other formats had a more uniform range of sizes.

Overall, compressed formats offer reasonable file sizes, but their compatibility with widely used open-source software remains a concern. For instance, the DEIMoS library only supports mzML and gzipped mzML, and MZmine only supports uncompressed mzML files (gzipped mzML files could not properly be opened). The reading speed of data stored in the Apache Parquet format was compared to that of data stored in mzML formats. Due to its tabular structure, a Parquet file could be opened directly as a pandas DataFrame (with the read_parquet() function) in 1–3 s, making it immediately available for processing with the DEIMoS library. In contrast, opening mzML files (with or without compression) with the DEIMoS library took between 15 and 20 min due to the parsing step required to convert the XML-structured data to a DataFrame. For comparison, parsing an uncompressed mzML file in MZmine took 1–2 min.

## 4 Integration and usage

While not directly compatible with software such as MZmine, the Apache Parquet format offers broad compatibility with multiple programming languages and development environments, including Java, C++, Python, R, or Julia. The simple tabular data structure of Parquet files facilitates their manipulation: they are quickly loaded as dataframes in programming environments (e.g. with the Arrow library in R or the pandas library in Python) and can then be easily filtered (e.g. with the dplyr syntax in the R environment; Wickham et al. 2023). The Arrow library supports on-disk filtration and access, allowing the manipulation of data without loading entire datasets into RAM (Richardson et al. 2024). Advanced filtering operations can be achieved with the DuckDB database system, which can query Arrow data directly with an SQL interface, without any data duplication (Mühleisen and Raasveldt 2024). This enables users to perform fast and complex queries that are not available within the Arrow library, even on files stored in cloud servers. The interoperability of Arrow, DuckDB, and dplyr makes queries easy to write in a few lines, providing user-friendly access to Parquet data, even for non-programmers and on computers with low resources. For example, the total ion chromatogram (sum of intensities for each retention time *rt*, for a given MS level) can be retrieved from a file (without loading it in RAM) using the following R code:

data = arrow :: open_dataset (“sample.parquet”)

TIC = data |>

  filter (mslevel == 1) |>

  group_by (rt) |>

  summarise(intensity = sum (intensity))|>

  collect ()

The same result can be achieved using Python (with data in RAM):

import pandas as pd

data = pd. read_parquet (“sample.parquet”, columns =[’rt’, ’mslevel’, ’intensity’])

ms1 = data [ data [’mslevel’] == ’1’]

TIC = ms1. groupby (’rt’, as_index = False).     sum (’intensity’)

The tabular data structure, whether as dataframes or Arrow data, also facilitates data analysis using popular signal processing packages, such as Scipy, for advanced statistical computations (e.g. machine learning on raw spectra). Additionally, data can be quickly visualized using standard graphics libraries (e.g. Matplotlib, Seaborn, Plotly, Bokeh). An R/Shiny companion application is provided to open and display HRMS data converted to Parquet files by the arcMS package. This application demonstrates how total ion chromatograms, base peak chromatograms, MS spectra, and 2D or even 3D plots can be efficiently displayed with the Arrow library without loading the entire dataset into RAM. These capabilities and the corresponding queries are detailed in the arcMS documentation.

HRMS Parquet files are also readily compatible with existing HRMS data processing pipelines using the same data structure, such as the DEIMoS Python library (Colby et al. 2022), a recent package designed to handle multidimensional HRMS data. DEIMoS provides features such as peak detection across multiple dimensions including IMS, data alignment across datasets and samples, as well as MS/MS spectra deconvolution. Integrating arcMS and DEIMoS into a pipeline offers an efficient method for processing HRMS data acquired with Waters UNIFI and including IMS. An automated and fully open-source workflow can thus be easily deployed for faster data analysis, such as the identification of environmental pollutants. Additionally, the compact file sizes of the Parquet format enable substantial storage savings and facilitate file sharing, which is particularly interesting for projects handling large data volumes.

## 5 Conclusions

The arcMS package facilitates the integration of HRMS data from the Waters UNIFI analytical platform into existing workflows or any programming environment. It uses the Apache Parquet format for lossless compression of raw data in profile mode, resulting in minimal file sizes with fast data accessibility. The compactness of the Parquet format is particularly beneficial for storing and sharing large volumes of HRMS data. Its columnar structure enables efficient manipulation of complex datasets with Arrow and DuckDB, even from distant servers, and ensures compatibility with novel tools like the DEIMoS library and potentially other tools based on columnar data across various programming languages. Although primarily designed for converting data from the Waters UNIFI platform and Waters (HD)MS^E^ data, the arcMS’s approach can be adapted to convert proprietary file formats from other vendors or techniques. For instance, Thermo RAW files can already be converted to the Parquet format (hulstaert2020j.proteomeres.). The broader adoption of open-source and efficient file formats, along with their integration into open-source tools and workflows, is promising for users of HRMS seeking greater control over their data and workflows.

## Data Availability

The data underlying this article are available in Software Heritage, and can be accessed at https://archive.softwareheritage.org/swh:1:dir:c6f1459ae4f2eb818064091c8e2e8248b9f5facc;origin=https://github.com/leesulab/arcMS;visit=swh:1:snp:8d989d471fd444985512e5412e3c16a0008f061c;anchor=swh:1:rev:2719d1a47cdd48b3221f1c11065cad6f313dd102, and in GitHub at https://github.com/leesulab/arcMS.
